# Management of a Complex Case of Gingival Enlargement Associated With Underlying Arteriovenous Malformation: A Five-Year Follow-Up Case Report

**DOI:** 10.7759/cureus.107795

**Published:** 2026-04-27

**Authors:** Nefeli Christou, Ioannis Michalis, Ioanna Mitsika, Spyridon Silvestros, Evangelia Zampa

**Affiliations:** 1 Dentistry, National and Kapodistrian University of Athens, Athens, GRC

**Keywords:** arteriovenous malformation, gingival enlargement, high-flow vascular anomalies, intraosseous arteriovenous malformation, vascular lesions

## Abstract

Arteriovenous malformations (AVMs) are high-flow congenital vascular anomalies characterized by abnormal direct communication between arteries and veins, bypassing the capillary bed. Gingival enlargement or gingival overgrowth is a common gingival disease characterized by an increase in the size of the gingival tissues. The main factors of gingival enlargement are inflammation, drugs, systemic conditions or diseases, neoplastic or false enlargements, and local factors, such as plaque, calculus, and/or overhanging restorations. The purpose of this article is to present an interesting case of the management of a patient with mandibular gingival enlargement occurring in association with an underlying AVM.

A 24-year-old non-smoker female patient presented to the periodontal clinic for the evaluation of a gingival overgrowth on the right side of the mandible that had developed over a four-week period, following a referral from her vascular specialist. Patient was previously diagnosed with an intraosseous AVMs in jaws (j-AVMs). Her medical history consisted of undergone embolization therapy for the j-AVM one year before. Clinical evaluation revealed an asymptomatic, soft-consistency, red/purple tumor-like mass with a broad base, measuring 0.5 x 0.5 cm. The surrounding gingival tissues in the right mandibular region appeared hyperplastic and edematous. A diagnosis of generalized gingivitis was established. The region of AVM was topographically associated with the vascular lesion of the mandibular gingiva. The gingival hyperplasia was distinguished from the underlying AVM, clarifying that the two represented coexisting independent lesions.

The coexistence of gingival overgrowth with an underlying intraosseous AVM is exceptionally uncommon and presents notable challenges in clinical management and treatment. This case highlights the importance of adopting a conservative and multidisciplinary management approach, particularly in patients with high-flow vascular anomalies, where invasive procedures may pose substantial risks. Effective management relies on close collaboration among dental clinicians, radiologists, and vascular specialists.

## Introduction

Vascular anomalies are soft-tissue lesions that are divided into two main categories: vascular neoplasms and vascular malformations (VMs). The main type of vascular tumor is a hemangioma, which is composed of rapidly proliferating endothelial cells. VMs are composed of abnormal, progressively enlarging vessels, which may involve arteries, veins, or lymphatic vessels. About 51% of VMs are localized in the head and neck region, and they occur more frequently in females, with a male-to-female ratio of 1:1.5 [1].

Arteriovenous VMs (AVMs) are high-flow vascular anomalies characterized by abnormal direct communication between arteries and veins, bypassing the capillary bed. AVMs are infrequent, accounting for about 1.5%, among vascular anomalies [2]. Although AVMs are generally congenital, they may enlarge when triggered by infections, trauma, or endocrine fluctuations. Due to their slow growth rate, AVMs often remain asymptomatic for extended periods and can present at any age. Intraosseous AVMs in jaws (j-AVMs) are very rare conditions; however, they carry a high risk of life-threatening bleeding, whether it occurs spontaneously or during surgery [3].

Gingival enlargement or gingival overgrowth is a common gingival disease characterized by an increase in the size of the gingival tissues. The etiology of gingival enlargement varies considerably, with the main factors including inflammatory, drug-influenced, those associated with systemic conditions or diseases, and neoplastic or false enlargements [4]. Local factors, such as plaque, calculus, and/or overhanging restorations, tend to be the most common underlying factors [5].

The purpose of this article is to present an interesting case of the management of a patient with mandibular gingival enlargement occurring in association with an underlying AVM.

## Case presentation

A 24-year-old non-smoker female patient, previously diagnosed with a j-AVM, presented to the periodontal clinic following a referral from her vascular specialist. She sought evaluation for gingival overgrowth on the right side of the mandible that had appeared four weeks before. Her medical history revealed that she had undergone embolization therapy for the j-AVM one year before her presentation. Notably, the patient was not receiving any systemic pharmacological treatment, thereby excluding drug-induced gingival enlargement from the differential diagnosis.

**Figure 1:**
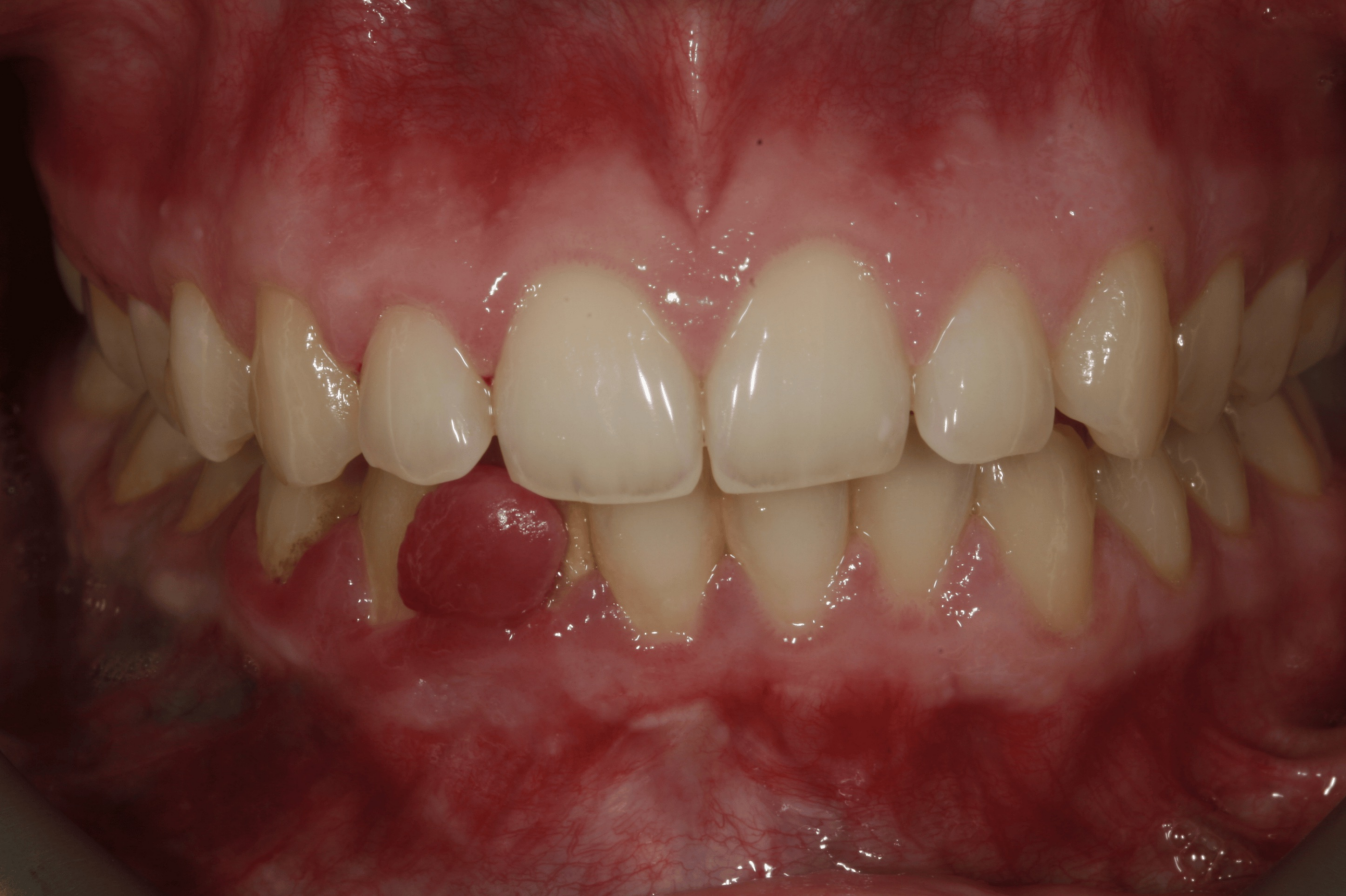
Initial intraoral image of the hyperplastic region of the mandibular

**Figure 2:**
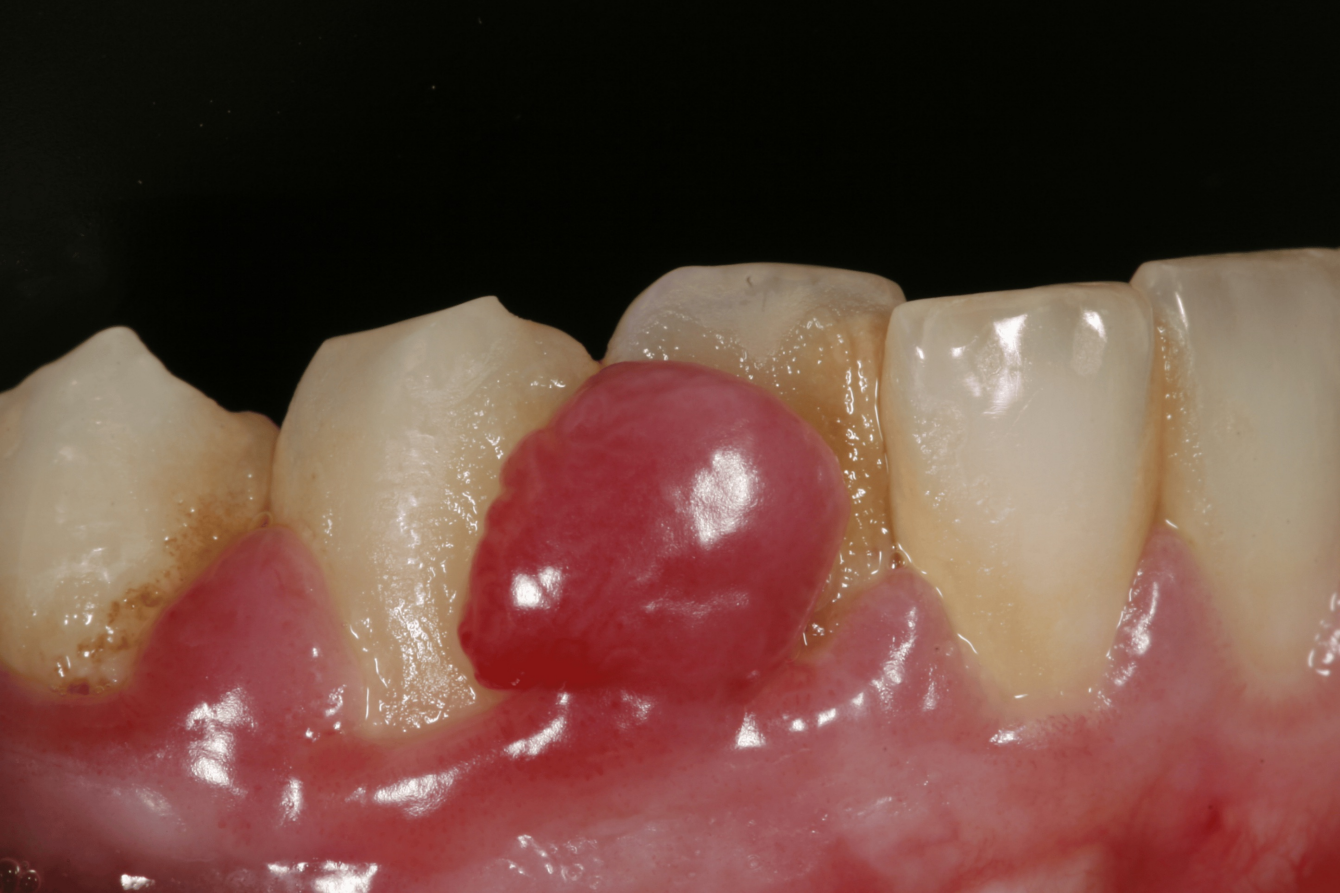
Initial intraoral image of the hyperplastic region of the mandibular

**Figure 3:**
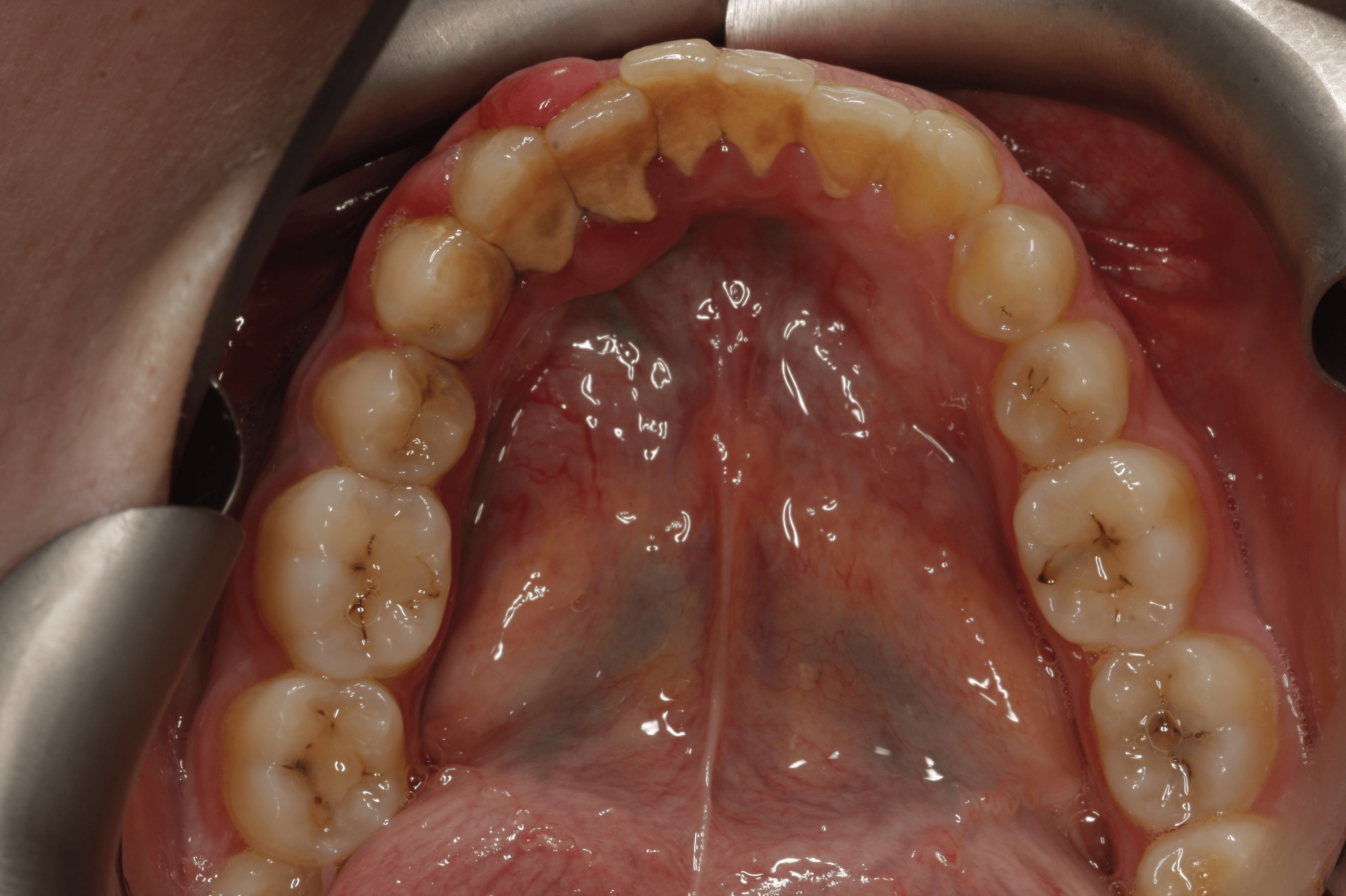
Initial intraoral image of the hyperplastic region of the mandibular

Extraoral examination revealed no significant findings. Intraoral evaluation revealed the main lesion: an asymptomatic, soft-consistency, red/purple tumor-like mass with a broad base, measuring 0.5 x 0.5 cm. This gingival hyperplasia presented initially as a small, painless lesion that had progressively enlarged to its current dimensions, extending between the right lateral incisor and the right canine on the labial mandibular gingiva. The surrounding gingival tissues in the right mandibular region appeared hyperplastic and edematous, closely associated with localized plaque accumulation (Figures 1-3).

No tooth mobility was noted. A comprehensive periodontal examination recorded bleeding on probing at ≥30% with no evidence of interdental attachment loss. Consequently, a diagnosis of generalized gingivitis was established. Given the patient’s known medical history, no other significant differential diagnostic concerns were initially identified.

**Figure 4:**
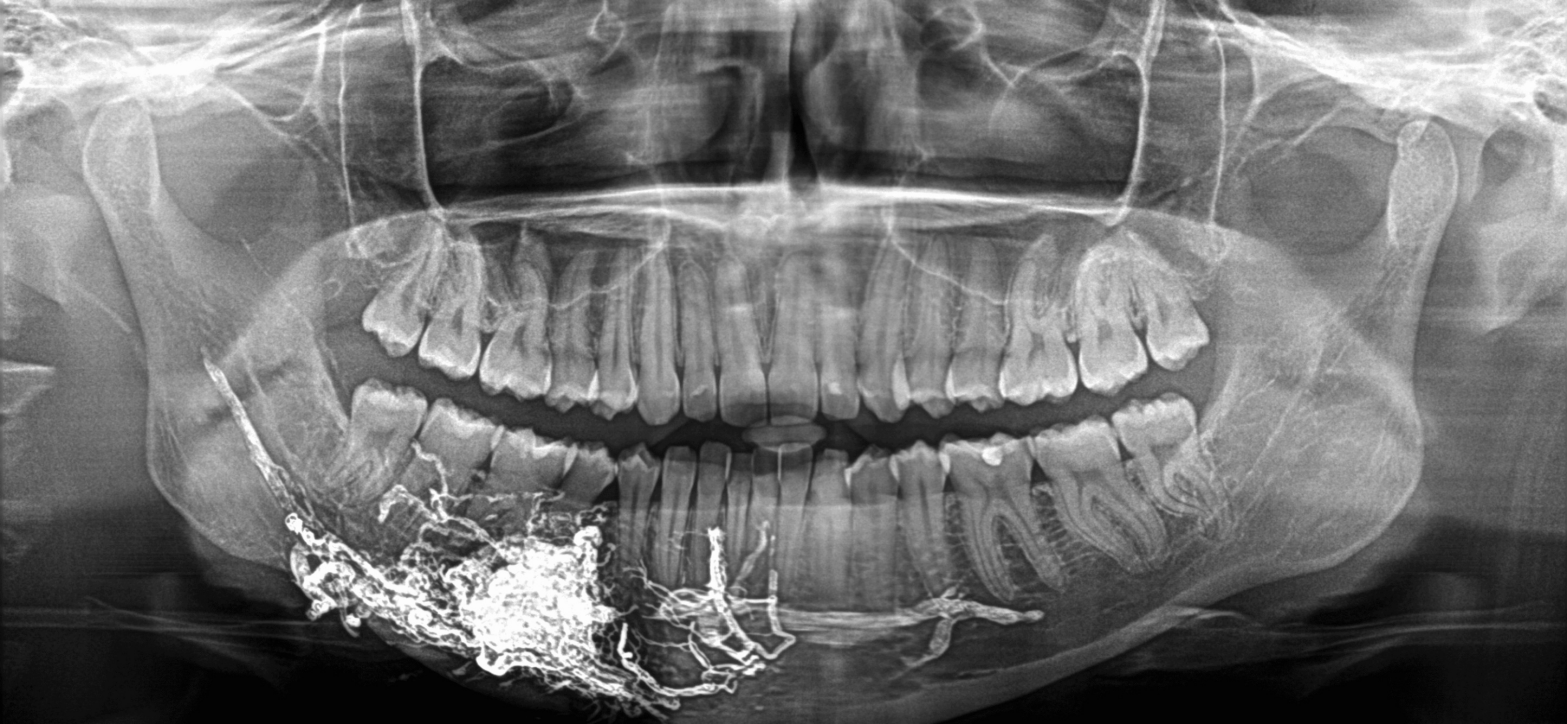
Panoramic radiograph and cone beam computed tomography (CBCT) revealed the embolic agent in the varix.

**Figure 5:**
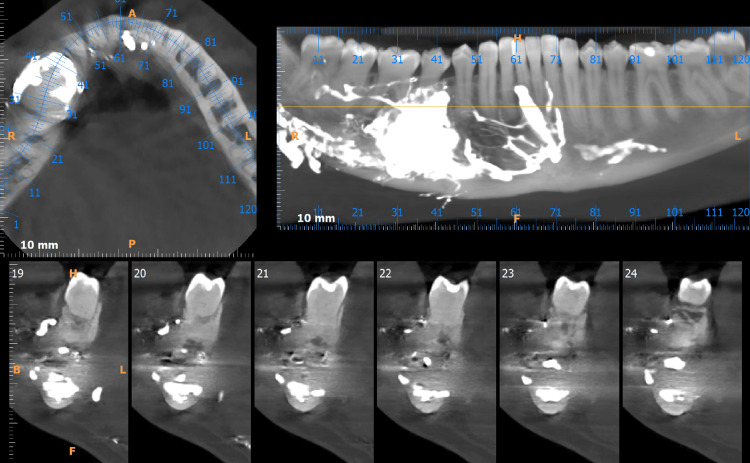
Panoramic radiograph and cone beam computed tomography (CBCT) revealed the embolic agent in the varix.

After radiographic examination, the size and location of the AVM were determined. Panoramic radiography and CT scan of the lower jaw revealed the intraosseous AVM located in the right side of the mandible. The region of AVM was topographically associated with the vascular lesion of the mandibular gingiva (Figures 4, 5).

**Figure 6:**
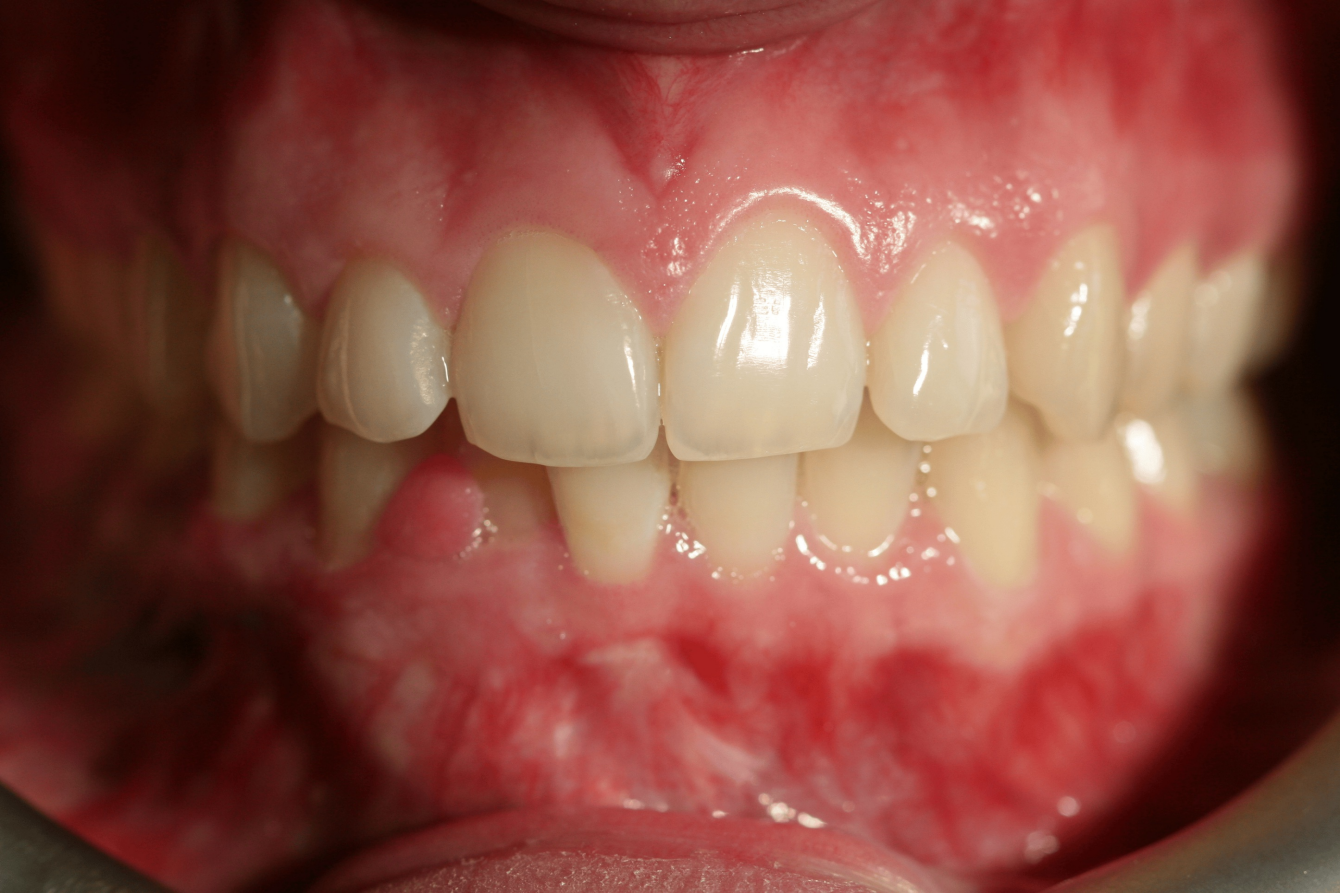
Reduction of the vascular lesion was observed four weeks after periodontal treatment and proper oral hygiene maintenance by the patient.

**Figure 7:**
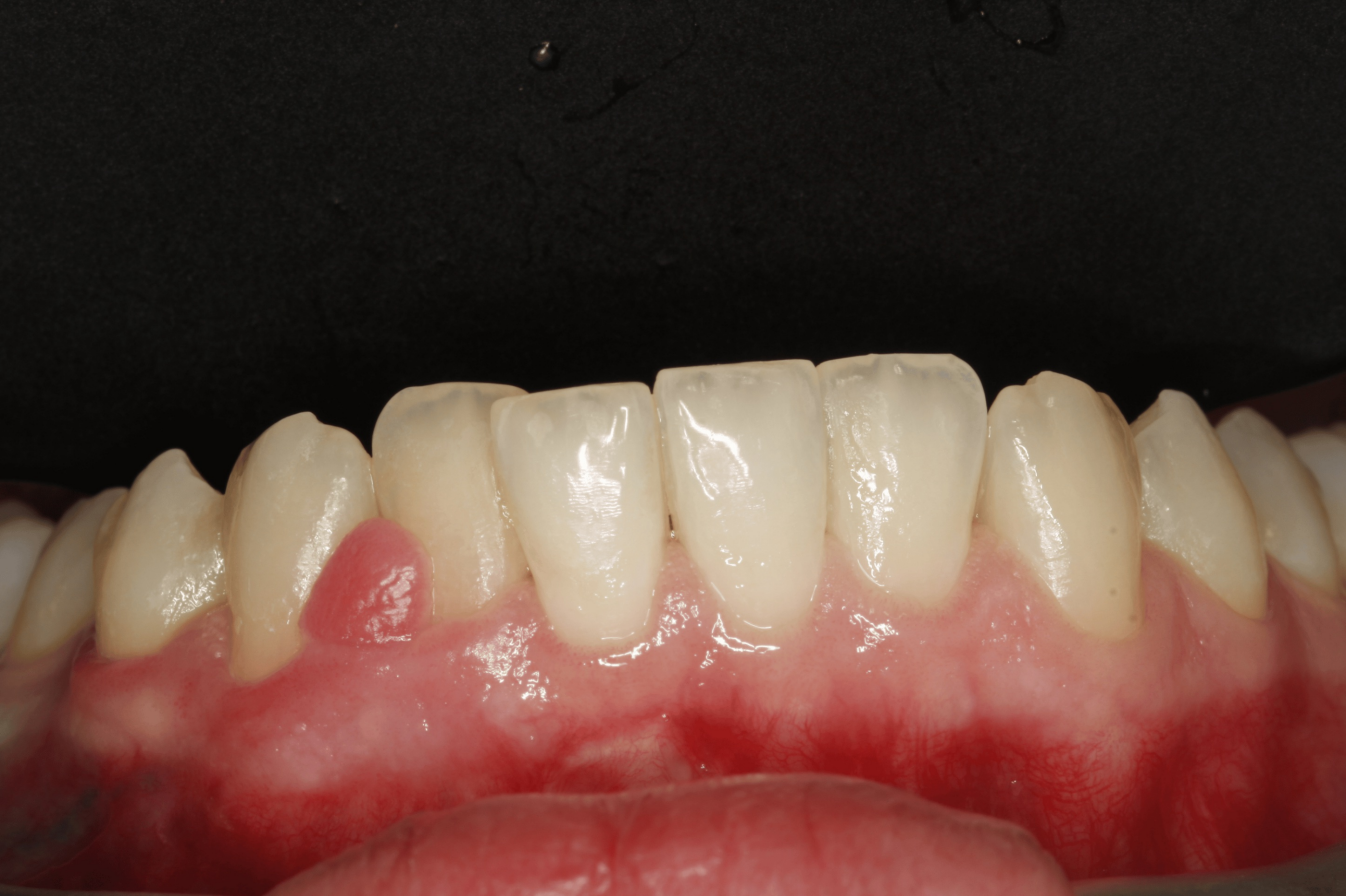
Reduction of the vascular lesion was observed four weeks after periodontal treatment and proper oral hygiene maintenance by the patient.

**Figure 8:**
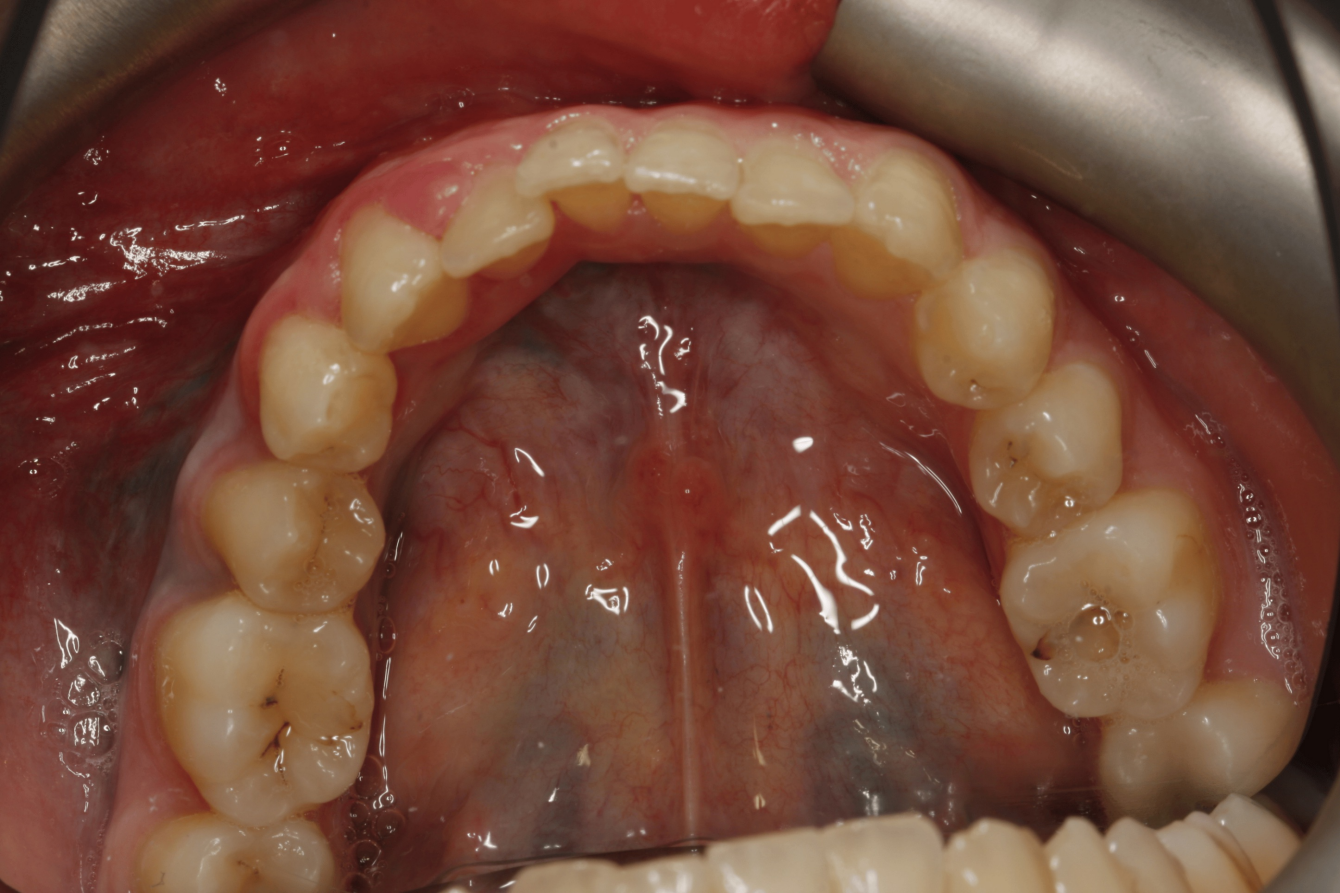
Reduction of the vascular lesion was observed four weeks after periodontal treatment and proper oral hygiene maintenance by the patient.

The gingival hyperplasia was distinguished from the underlying AVM, clarifying that the two represented coexisting independent lesions. The extremely rare coexistence of gingival enlargement and intraosseous AVM poses a significant challenge for clinical management and treatment. A conservative approach was initially adopted, comprising mechanical debridement to eliminate local microbial factors, coupled with tailored oral hygiene instructions. Collaboration with patient’s vascular specialist was essential. During the four-week follow-up examination, the clinical appearance of the area was markedly improved, showing complete reduction of the lesion and clinical signs consistent with lack of inflammatory activity (Figures 6-8).

**Figure 9:**
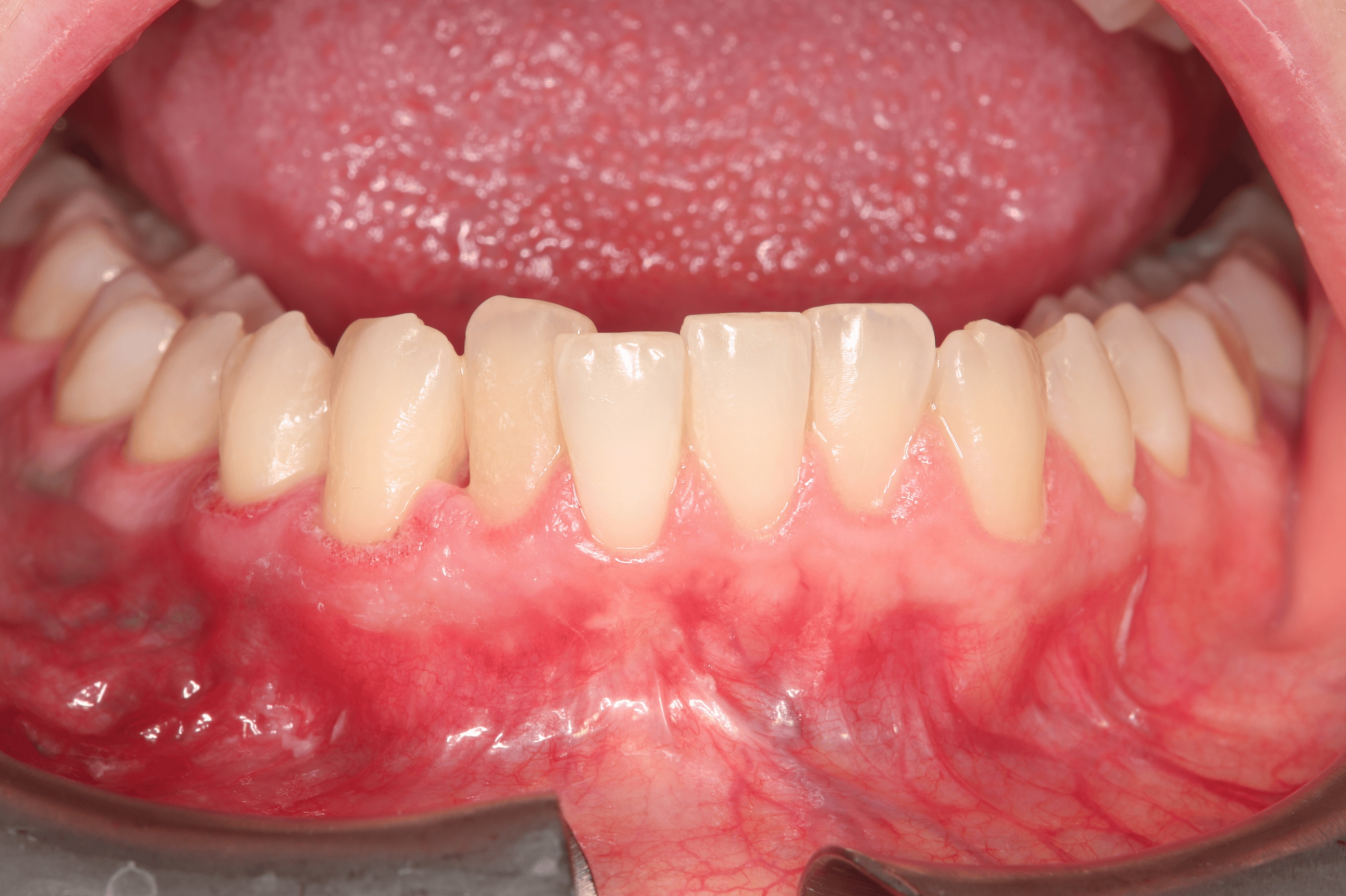
Image right after the removal of hyperplastic tissues using ER: YAG laser technology (200 mJ, 18 Hz)

**Figure 10:**
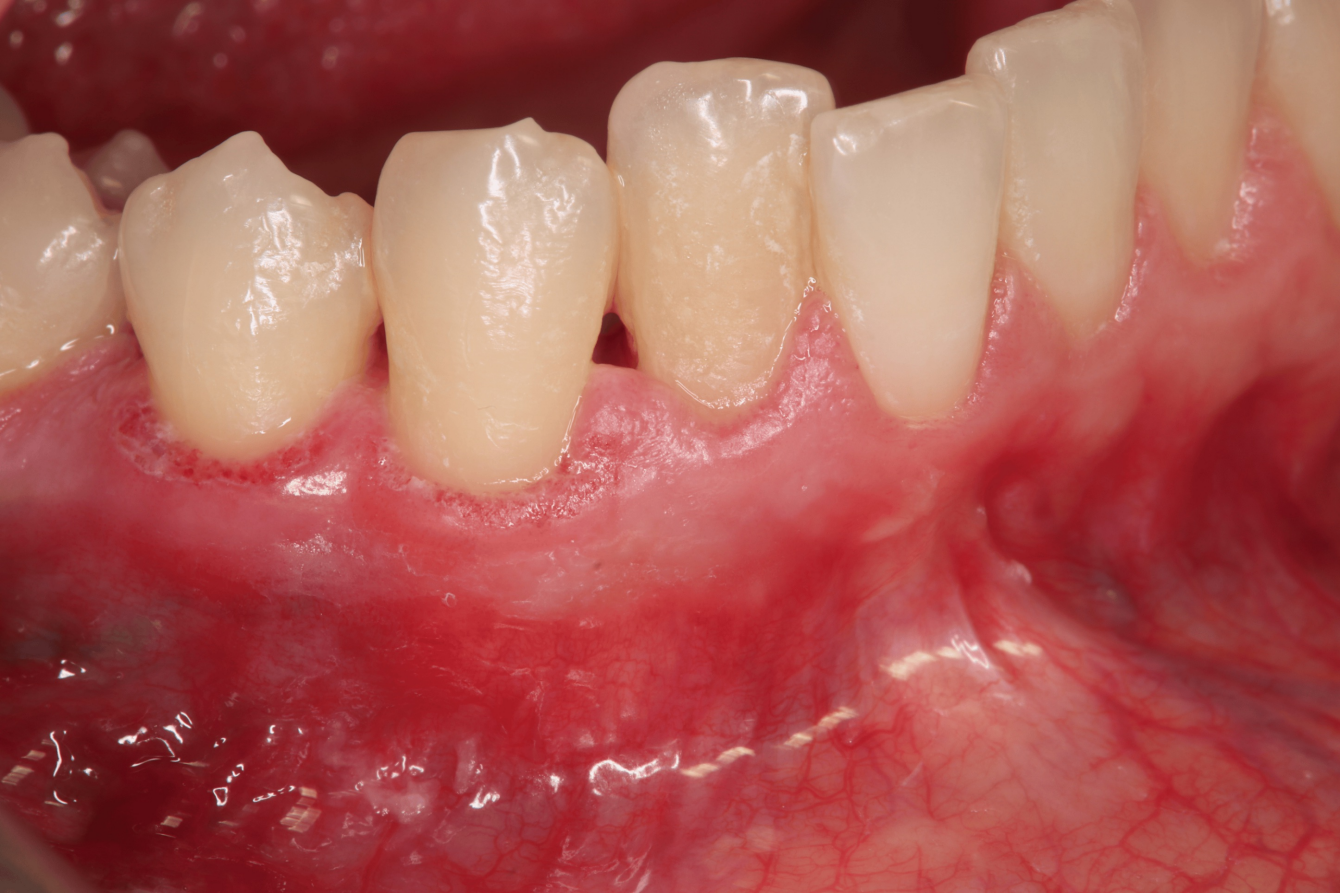
Image right after the removal of hyperplastic tissues using ER: YAG laser technology (200 mJ, 18 Hz)

Reduction of the lesions and inflammatory signs enabled surgical intervention in the region for the removal of the remaining hyperplastic tissue, using Er: YAG laser at 200 mJ and 18 Hz, minimizing the risk of uncontrolled bleeding during or after surgery (Figures 9, 10).

The excised hyperplastic tissue was submitted for histopathological examination (Supplementary Table 1). The microscopic analysis revealed features consistent with reactive/inflammatory gingival hyperplasia, showing no evidence of VM, dysplasia, or neoplastic alterations within the specimen. The histopathological report definitively confirmed our clinical hypothesis. It successfully distinguished the reactive gingival overgrowth from the underlying j-AVM, proving that the two represented coexisting but pathologically independent lesions.

**Figure 11:**
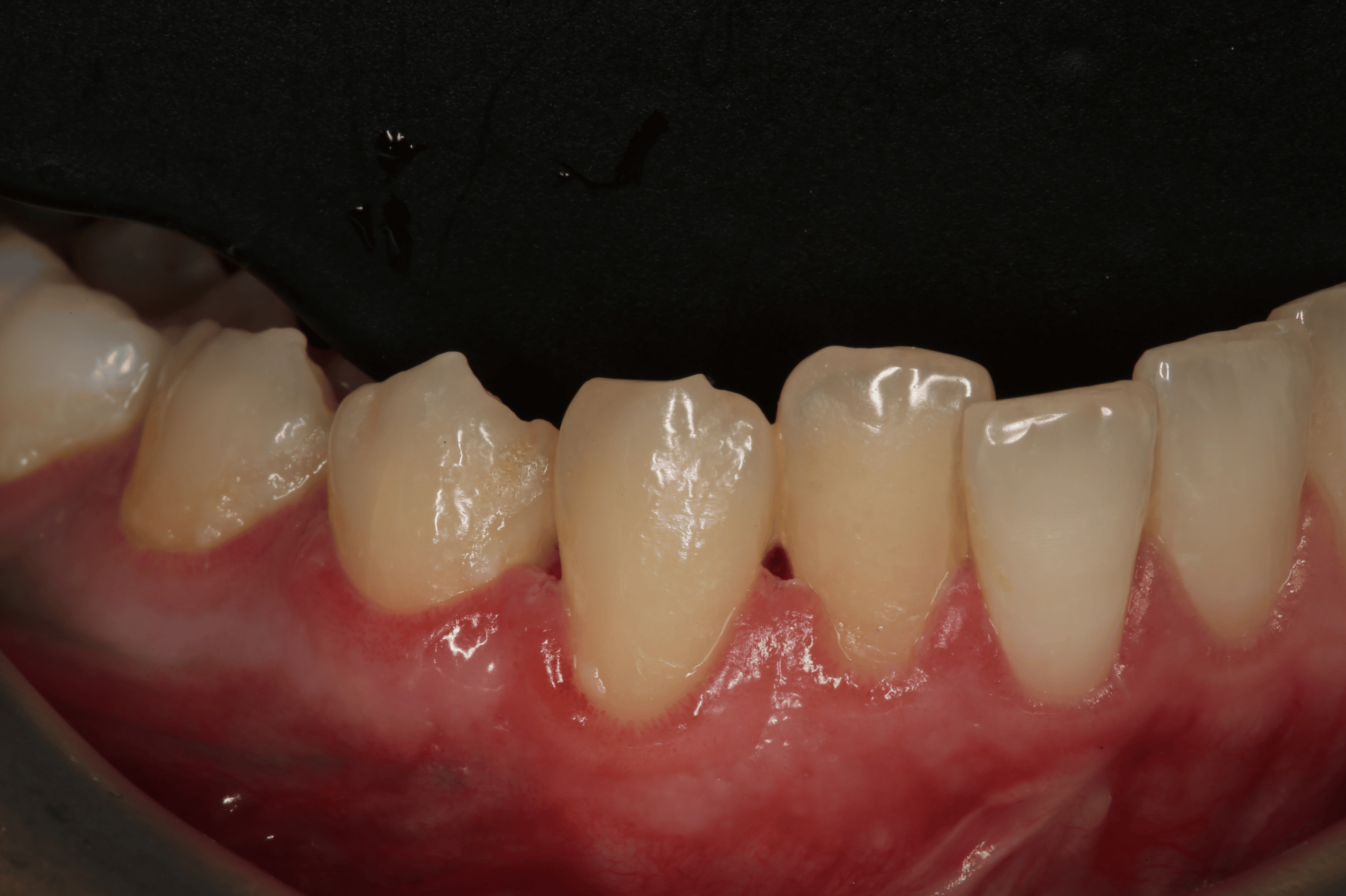
Image five years after the treatment showed no evidence of inflammatory activity or recurrence of the lesion.

The patient was enrolled in a strict recall schedule and supported oral hygiene protocols, despite her initial concerns regarding brushing the affected region. Follow-up evaluations every six months showed no evidence of inflammatory activity or recurrence of the lesion even after five years (Figure 11).

## Discussion

AVMs are congenital vascular anomalies in which arteries connect directly to veins through a tangled network of abnormal vessels (nidus), without the normal capillary bed in between. The low blood resistance of the nidus increases blood flow. The increased blood pressure and speed make the outflow vein dilated and arterialized [2].

Histologically, AVMs consist of multiple abnormal arteriovenous shunts lacking a normal intervening capillary network. Vessels are composed of arterial and venous components with no proper muscular walls and show no evidence of endothelial proliferation or giant cell formation [6]. The etiology of AVMs is generally attributed to environmental factors and traumas leading to vascular rupture. In approximately 90% of cases, arteriovenous lesions can have a traumatic or congenital origin [7].

The clinical presentation of AVMs can vary widely depending on the location, size, and extent of the lesion. Intraoral lesions can impair speech, mastication, and swallowing. Additionally, when AVMs extend intraosseously, they can lead to gingival bleeding, tooth mobility, and occlusal discrepancy [8]. The most common symptom of j-AVMs is gingival bleeding and uncontrollable hemorrhage in the alveolar socket. Management of bleeding is the most essential step in the treatment of AVMs. Digital subtraction angiography (DSA) is used not only for the diagnosis of AVM but also for blocking the supplying artery to reduce vascularity [9].

The radiologic features of AVMs include bone erosion and irregular radiolucent areas. CT/MRI reveals irregular margins, enhancement of the cancellous bone, and the presence of enlarged vessels. DSA reveals the characteristic features of AVMs and the abnormal direct arteriovenous communication [10]. Accurate diagnosis is essential for the proper management of VMs; DSA remains the gold standard diagnostic method [11]. According to histopathological analysis, VMs are classified based on the type of vessels that appear to be malformed (capillary, lymphatic, venous, arteriovenous, and combined malformations) [12]. Valid differential diagnosis of vascular lesions is crucial for correct and effective therapeutic management. Surgical removal of vascular tumors can be performed with a relatively low risk of complications, while VMs can cause uncontrolled life-threatening hemorrhage during surgery [13].

Clinical management and treatment of AVMs lack a standardized protocol, with current approaches varying widely. While sclerotherapy and embolization are most frequently reported as primary treatment modalities, the published literature shows a lack of methodological uniformity [14]. Therapeutic success relies on achieving nidus occlusion, as the nidus underlies lesion recurrence. However, this remains challenging due to the complex arterial architecture of high-flow AVMs. Similarly, embolization also proves challenging, with no fully effective and safe embolic agent yet developed. Overall, treatment of high-flow AVMs carries a significant risk of recurrence, highlighting the necessity for additional studies for the development of an effective standardized therapeutic strategy [15].

Gingival overgrowth compromises oral hygiene maintenance, which secondarily exacerbates the inflammatory aspect, exaggerating the existing condition and potentially affecting speech, mastication, and esthetics. Gingival enlargement can range from mild enlargement of isolated interdental papillae to a uniform marked enlargement involving either one or both jaws. It is a common clinical finding that primarily represents reactive hyperplasia due to plaque-induced inflammatory gingival disease [16]. Clinically, inflammatory gingival hyperplasia presents as soft, edematous tissues with marked color changes, typically characterized by a pronounced erythematous appearance. Spontaneous bleeding is frequently observed. Initial management involves scaling and root planning with complete removal of plaque and calculus deposits, resulting in partial or complete resolution of the tissue enlargement [17,18]. In cases where the tissue enlargement only partially resolves, the use of laser surgery for the excision of the hyperplastic tissue offers significant advantages. Primarily, its excellent hemostatic properties minimize the risk of intraoperative and postoperative bleeding. Additionally, it provides precise tissue ablation with minimal thermal damage to surrounding structures, thereby promoting faster healing [19].

## Conclusions

The present case highlights the diagnostic and therapeutic challenges associated with the uncommon coexistence of gingival enlargement and an underlying mandibular AVM. Careful clinical evaluation, appropriate imaging, and histopathological confirmation are essential to establish an accurate diagnosis and to differentiate AVMs from other vascular or reactive lesions of the oral cavity. This case underlines the importance of adopting a conservative and multidisciplinary management approach, particularly in patients with high-flow vascular anomalies, where invasive procedures may pose substantial risks. Collaboration between dental clinicians, radiologists, and vascular surgeons is crucial to ensure safe and effective treatment. Early recognition of vascular lesions and individualized management strategies can prevent serious complications and contribute to improving the patient’s oral health.

## Appendices

**Table 1:** Histopathological report data of the excised gingival tissue

Parameter	Description
Specimen	Gingival tissue specimen
Macroscopic description	Soft-consistency, red/purple tumor-like mass with a broad base, measuring 0.3 x 0.3 cm
Microscopic findings	Features consistent with reactive/inflammatory gingival hyperplasia. There is no evidence of vascular malformation, dysplasia, or neoplastic alterations within the specimen
Final diagnosis	Reactive/inflammatory gingival hyperplasia
